# R-(-)-carvone Attenuated Doxorubicin Induced Cardiotoxicity *In Vivo* and Potentiated Its Anticancer Toxicity *In Vitro*

**DOI:** 10.4274/balkanmedj.galenos.2019.2019.7.117

**Published:** 2020-02-28

**Authors:** Manal Mohammad Abbas, Yasser İbrahim Kandil, Manal Ahmad Abbas

**Affiliations:** 1 School of Allied Medical Sciences, Al-Ahliyya Amman University, Amman, Jordan; 2 School of Pharmacy, Al-Ahliyya Amman University, Amman, Jordan; 3 School of Pharmacy, Al-Azhar University, Cairo, Egypt

**Keywords:** Cardiotoxicity, catalase, doxorubicin, H9C2, MCF 7, R-(-)-carvone

## Abstract

**Background:**

Doxorubicin is one of the most potent broad-spectrum antitumor and chemotherapeutic agents. However, it produces cardiotoxicity.

**Aims:**

To investigate whether R-(-)-carvone exerts a cardioprotective effect against doxorubicin toxicity *in vivo* and *in vitro*.

**Study Design:**

Cell culture and animal experiment.

**Methods:**

The synergistic effect of R-(-)-carvone with doxorubicin was evaluated in the MCF 7 cancer cell line while its protective effect against doxorubicin toxicity was evaluated in the normal heart cell line (H9C2) and *in vivo*. Furthermore, the mechanism of its cardioprotective effect was studied.

**Results:**

R-(-)-carvone exerted cytotoxic action on the MCF 7 cancer cell line with an IC_50_ value of 14.22 μM and potentiated the cytotoxic action of doxorubicin, while it decreased the toxicity of doxorubicin on a normal heart cell line. In BALB/c mice, R-(-)-carvone protected the heart from the toxic action of doxorubicin, as was evident by biochemical and histological studies. The protective effect of R-(-)-carvone on the H9C2 heart cell line and on heart *in vivo* was due to an increase in catalase activity.

**Conclusion:**

R-(-)-carvone has synergistic anticancer action with doxorubicin on the MCF 7 cell line while decreasing its cardiotoxicity.

Doxorubicin (DOX), an anthracycline antibiotic, is one of the most potent broad-spectrum antitumor and chemotherapeutic agents used since late 1960s (1). Unfortunately, the cytostatic action of DOX in therapeutic doses is insufficient in many cases; therefore, higher doses of DOX are used for treatment. As a result, systemic toxicity, particularly cardiotoxicity, develops (2).  A previous research aims at increasing DOX efficacy in tumor cells while minimizing its associated toxicities to non-cancerous tissues (3). A variety of approaches have been examined to minimize the effective chemotherapeutic dose of DOX and, thereby, its side effects. One of these approaches is the use of natural compounds with anticancer or chemopreventive properties that can be used in combination with DOX (4). Another approach is the use of natural antioxidants that can ameliorate DOX-induced cardiotoxicity by reducing oxidative stress (5).

Carvone belongs to volatile monocyclic terpenoid that is a constituent of many essential oils, but it is mostly concentrated in caraway *(Carum carvi)*, dill *(Anethum graveolens)* and spearmint *(Mentha spicata)* seed oils (6). This monoterpene is present as two enantiomers that differ in their biological properties. These enantiomers are S-(+)-carvone (D-carvone) present in caraway seeds and R-(-)-carvone (L-carvone) present in mint leaves (6). R-(-)-carvone was used as an effective chemopreventive agent for colon carcinogenesis (7). Also, 4S-(+)-carvone caused about a 60% reduction in forestomach tumors and nearly a 35% decrease in pulmonary adenoma formation in female A/J mice by inhibiting N-nitrosodiethylamine-induced carcinogenesis (8).

The protective effect of carvone against paclitaxel-induced retinal and optic nerve cytotoxicity was reported recently (9). However, its protective effect against DOX-induced cardiotoxicity was not studied yet. Therefore, this study was designed to investigate the cardioprotective effect of R-(-)-carvone in a normal heart cell line (H9C2) *in vitro* and BALB/c mice *in vivo*. Also, the synergistic cytotoxic effect of R-(-)-carvone with DOX on the MCF 7 cell line was investigated.

## MATERIALS AND METHODS

### *In vivo* study: Cardioprotective effect of R-(-)-carvone against DOX toxicity in mice

**Figure 1:**
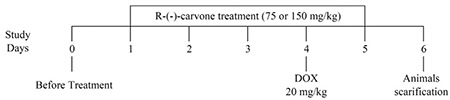
Scheme for experimental design. DOX: Doxorubicin

The study was conducted in the Experimental Animal Laboratory of the Faculty of Pharmacy, Al-Ahliyya Amman University. The ethics committee approved all submitted for this study (ethical approval number AAU-2/5/2018) at Al-Ahlyyia Amman University. For acclimatization purposes, mice were kept one week before the experiments under the standard environmental conditions (temperature 23+2 °C, 12 h dark/light period). Male BALB/c (Bagg albino) mice were divided into four groups, each containing eight animals weighing between 23 and 28 g. Group I was the negative control group that received vehicle (2% Tween 20) only. Group II was the positive control that received vehicle and DOX (Sigma, USA) dissolved in sterile normal saline. Groups III and IV were treatment groups that received R-(-)-carvone (Sigma, USA) (75 and 150 mg/kg suspended in 2% Tween 20), respectively. Vehicle and R-(-)-carvone were given intraperitoneally (i.p.) once daily for five consecutive days (Figure 1). DOX (20 mg/kg) was given to groups II, III, and IV as a single i.p. injection one hour after receiving treatment (vehicle or R-(-)-carvone) on day 4 of the experiment. Animals were euthanized on day 6 (48 h after DOX) under light ether anesthesia followed by cervical dislocation. The choice of R-(-)-carvone dose was based on previously conducted pilot studies, while the choice of DOX dose was based on previous studies (10). The DOX dose used produced 0% mortality with histological and biochemical changes evident for DOX cardiotoxicity.

### Collection of blood samples and tissue preparation

After the designated period, blood samples were obtained from the retro-orbital plexus using glass heparinized capillary tubes. Sera were separated and stored at -20 °C until biochemical analyses. Hearts were obtained immediately after sacrifice and washed several times with ice-cold saline. Sections of the heart were fixed in 10% buffered formalin for histopathological study, and the remaining heart tissue was stored at -80 °C until homogenization.

### Heart tissue homogenization

About 100 mg of heart tissue was homogenized in ice-cold (1:9 wt/vol) cell lysis buffer (RnD, Cat. #: 895347, USA) with a Teflon homogenizer (Potter-Elvehjem). The homogenate was centrifuged at 12,000 g for 10 min at 4 °C. The collected supernatants were collected and kept at -80 °C until they were analyzed for catalase activity. Protein concentrations of supernatants were assayed by the Lowry method (11).

### Histopathological examination

The formalin-fixed tissues were embedded in paraffin, sectioned at 5 µm and the sections, and then stained with eosin/hematoxylin. Sections of the heart from each mouse in all groups were examined and photographed using a light microscope MC 170 HD Leica Camera, Switzerland. All the qualitative histological analysis was performed by one of the authors (M.A.A.)

### Biochemical tests

Total creatine kinase (CK), (Biosystems Cat. #: 11790) and lactate dehydrogenase (LDH) (Biosystems, Cat. #: 11580) activities in serum were assayed spectrophotometrically according to the manufacturer’s guidelines.

### *In vitro* study: Cell viability assay

The MCF 7 (an invasive breast ductal carcinoma) and H9C2 (normal cardiomyocyte) cell lines were obtained from The European Collection of Authenticated Cell Cultures (UK). The cell lines were grown in a humidified 5% CO2 atmosphere incubator at 37 °C and in DMEM high glucose (Euroclone, S.p.A) containing 10% fetal bovine serum, 10 g/L penicillin-streptomycin and 10 g/L L-glutamine. A stock solution of R-(-)-carvone and DOX (1:1 ratio) was freshly prepared in 2-fold serial dilutions. A stock solution of each agent was prepared in dimethyl sulfoxide (0.5%). The effect of R-(-)-carvone and DOX combination on the MCF 7 and H9C2 cell lines was evaluated by MTT (3-(4,5-dimethylthiazol-2-yl)-2,5-diphenyl-tetrazolium bromide) assay (Promega, USA). Cells were seeded into 96-well plates at a density of 7000 cells/well in a suitable medium. After 24 h incubation at 37 °C, cells were treated with R-(-)-carvone, DOX, or their (1:1) combination in serial dilution ranging from 100 µM to 6.25 µM for 48 h. MTT was then performed using an MTT kit where 15 µL of the staining reagent was added to each well, and incubated for four h at 37 °C followed by the addition of 100 µL of the solubilization stop/mix solution and incubation for 1 h before measuring the absorbance using microplate reader (Biotech, USA) at 590 nm.

### Synergy

Synergy evaluation was performed for R-(-)-carvone in combination with the anticancer drug (DOX). It was performed against the MCF 7 and H9C2 cell lines using the MTT test as described above. The degree of synergism was determined by calculating the Combination index (CI) using Compusyn software (version 1.0) based on the median-effect analysis of Chou and Talalay given that CI<1, CI=1, and CI>1 indicates synergy, additive, and antagonism, respectively (12).

### Antioxidant test (catalase) assay

For cultured cells, the cells were harvested and homogenized with cell lysing buffer (Cat. #: 895347, R&D Systems Inc. Minneapolis, USA) and sonicated in ice three cycles, 90 s/cycle using a sonicator (TI-H-5 Elma, Schmidbauer GmbH, Germany). The catalase (Cayman Cat. #: 707002, USA) test was performed according to the manufacturer’s directions.

### Statistical analysis

All data passed the normality test (Shapiro-Wilk). Values were presented as mean ± standard error except for IC50 experiment where values were represented as mean + standard deviation. Differences between group means in CK, LDH, and catalase were analyzed by one-way analysis of variance and post-hoc Turkey’s test using GraphPad Prism version 6 (GraphPad Software, San Diego, USA). Results were considered statistically significant when p<0.05.

## RESULTS

### *In vivo* study

**Figure 2:**
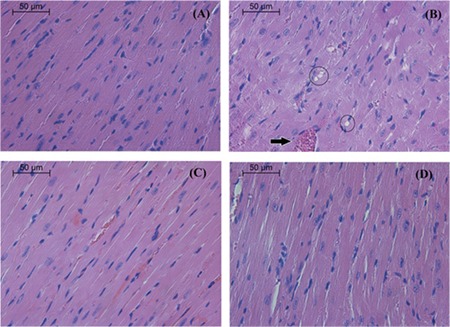
Histology of heart tissue from mice receiving only vehicle (A), DOX (B), R-(-)-carvone (75 mg/kg) plus DOX (C), and R-(-)-carvone (150 mg/kg) plus DOX (D). Note myocardial fiber injury in B represented by vacuolation of the cytoplasm (circles). Also, congestion in blood vessels is obvious (black arrow). R-(-)-carvone 150 mg/kg in D protected the heart from the effects of DOX (H&E stain). DOX: doxorubicin

On histopathological analysis, the heart tissue of mice that received DOX at a dose of 20 mg/kg showed disorganization and degeneration of the myocardium. Concomitant treatment with R-(-)-carvone revealed normal muscle fiber architecture (Figure 2). DOX treatment increased CK and LDH activities in serum (Table 1). Only the high dose of R-(-)-carvone (150 mg/kg) was able to lower the activities of both enzymes significantly in animals that received R-(-)-carvone along with DOX.

### *In vitro* study

**Figure 3:**
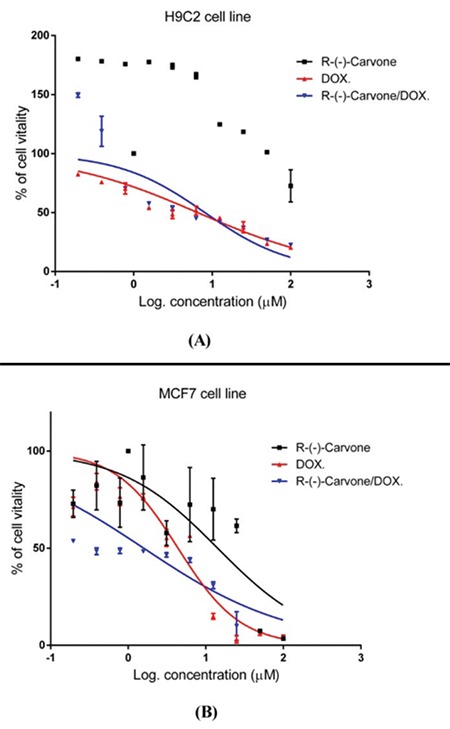
Cytotoxic effect of R-(-)-carvone alone or in combination with Dox in H9C2, and MCF 7 cell lines. Carvone decreased the cytotoxicity of DOX in H9C2 cells (A) and enhanced the cytotoxicity of DOX in MCF 7 cells (B). The IC50 value of all treatments was measured by the MTT assay. DOX: doxorubicin

The half-maximal inhibitory concentration (IC_50_) values of R-(-)-carvone, DOX, and the DOX and R-(-)-carvone combination (1:1) in H9C2 and MCF 7 cell lines are illustrated in Table 2. R-(-)-carvone was non-toxic up to 200 μM on H9C2 normal heart cells while it exerted cytotoxic action on the MCF 7 cancer cell line with IC_50_ values of 14.22 µM. Using CompuSyn software, R-(-)-carvone/DOX exerted antagonistic effect on H9C2 cells, but a synergistic effect on the MCF 7 cell line (Table 2). R-(-)-carvone potentiated the cytotoxic action of DOX on the MCF 7 cell line, whereas it decreased the toxicity on the H9C2 (Figure 3).

### Catalase assay

**Figure 4:**
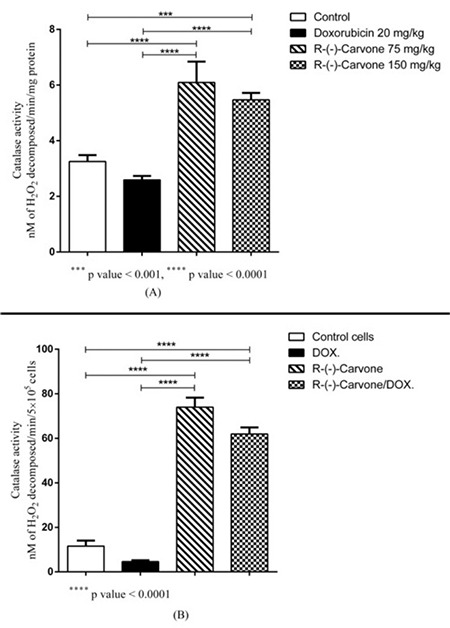
Results of catalase activity in normal heart in vivo. Catalase activity was increased by R-(-)-carvone alone or by R-(-)-carvone/DOX combination in normal heart in vivo (A). Catalase activity in in the heart cell line (H9C2). Both doses of R-(-)-carvone alone and the combination of R-(-)-carvone with DOX increased catalase activity in the heart cell line (B). DOX: doxorubicin

Catalase activity was increased by R-(-)-carvone alone or by R-(-)-carvone/DOX combination in normal heart *in vivo* (Figure 4A). Similarly, both doses of R-(-)-carvone alone and the combination of R-(-)-carvone with DOX increased catalase activity in the H9C2 (Figure 4B).

## DISCUSSION

In the present investigation, the synergistic effect of R-(-)-carvone on DOX cytotoxicity was investigated in the MCF 7 breast cancer cell line. The MCF 7 cell line was chosen because DOX is widely used for treatment of breast cancer. However, the use of DOX is limited by its cardiotoxicity (13). Therefore, the protective effect of R-(-)-carvone on the normal H9C2 was studied.

In the present study, R-(-)-carvone had cytotoxic effect on the breast adenocarcinoma cell line with an IC_50_ value of 14.22 µM. Other studies reported IC_50_ values of 166.2 µM (14), 1.2 mM (15), and 0.63 µM (16) in the same cell line. The differences in IC_50_ values could be due to the use of different carvone isomers in different studies, the use of different methods for assessing the cytotoxicity and/or different growth conditions.

The critical finding of this study is that R-(-)-carvone exerted a synergistic cytotoxic effect when administered with DOX on MCF 7, whereas its effect was antagonistic on the H9C2. This indicates that R-(-)-carvone can decrease the toxicity on normal heart cells while increasing it in the tested cancer cell line *in vitro*. Similarly, the protective effect of R-(-)-carvone *in vivo* was evident on the heart by lowering the elevated activities in CK and LDH caused by DOX treatment. Also, the histological degenerative changes produced by DOX were less in R-(-)-carvone-treated groups. It should be noted that only males were used in this study because the male sex hormone level is almost constant, while that of female sex hormones vary along the estrous cycle. The results of the present work may differ if females were used (17). Therefore, it would be interesting to investigate the effect of R-(-)-carvone in females in future studies.

The findings of this study raise a question: Why did R-(-)-carvone exert a synergistic effect with DOX on the MCF 7 cell line while it produced an antagonistic effect on the normal heart cell line? The mechanism of the R-(-)-carvone antagonistic effect was not investigated herein. However, previous study have reported that R-(-)-carvone induces p53, caspase 3-mediated apoptosis in a breast cancer cell line, and arrests MCF 7 cells in the S phase of the cell cycle (15). Another proposed mechanism for R-(-)-carvone action in MCF 7 cells is that L-carvone (R-(-)-carvone) inhibits the enzyme poly ADP ribose polymerase (PARP) (15). Several forms of cancer are more dependent on PARP than healthy cells, thereby making PARP an attractive target for cancer therapy.

Different mechanisms were proposed to explain DOX anticancer action. These include targeting the enzyme topoisomerase II, which is essential in DNA replication and iron chelation (15). High levels of topoisomerase II is usually present in cancer cells, especially solid tumors like breast cancer, because they proliferate quickly (15). Another proposed mechanism for DOX anticancer action is iron chelation. Iron is important in cell proliferation, DNA synthesis, mitochondrial electron transport, and oxygen sensing. Due to high rate of proliferation in cancer cells, more demand for iron exists. Therefore, by chelating iron, DOX slows down the growth rate of cancer cells. Unfortunately, this process generates iron-mediated reactive oxygen species (ROS), which may cause cardiomyopathy (15).

The ROS elevation is a therapeutic approach used in cancer treatment. Increasing the endogenous ROS threshold level in cancer cells might also put healthy cells of some organs that are vulnerable to oxidative toxicity, such as kidney liver and heart (18). The most frequently proposed mechanism to explain the complex pathophysiology of DOX-induced cardiotoxicity is oxidative stress (19,20). Therefore, current research aims at identifying pharmacological agents that may enhance oxidative stress in cancer cells and protect healthy cells from oxidative damage (18). It has been recently demonstrated using microarray analysis that DOX changed the expression of genes involved in the oxidative stress pathway (21).

In the present study, R-(-)-carvone exerted its protective effect by increasing catalase activity in the H9C2 *in vitro* as well as in the hearts of mice *in vivo*. Catalase is considered one of the major enzymes involved in the detoxification of hydrogen peroxide (H_2_O_2_). Overexpression of catalase in the heart of transgenic mice suppressed DOX cardiotoxicity (22). The antioxidant effect of R-(-)-carvone was demonstrated previously *in vitro* (14,23), whereas D-(+)-carvone also increased catalase activity *in vivo* (7). A recent study demonstrated that carvone exerted a protective effect against paclitaxel-induced retinal and optic nerve cytotoxicity by counteracting oxidative stress (9).

The use of antioxidant in cancer therapy is controversial. In one way, antioxidants may play important role in reducing the severity of a drug’s adverse effects. However, other antioxidants may interfere with DOX therapeutic effectiveness (3) since the cytotoxic action of DOX involves oxidative stress and the production of free radicals (5). Clinical and experimental studies have shown that dietary antioxidants not only reduce the adverse effects of anticancer agents but also increase the efficacy of conventional cancer therapy. However, some reports supported that antioxidant supplements during cancer treatments reduce the effectiveness of anticancer therapy (18).

Several studies have reported that natural products are effective in decreasing the toxicity of DOX by enhancing antioxidant defenses. For example, the monoterpene D-limonene suppressed DOX-induced oxidative stress and inflammation in the kidneys of Wistar rats (24). Similarly, the monoterpene geraniol had protective effect on ventricular cardiomyocytes of neonatal rats that were subjected to oxidative stress (25). Also, hesperetin ameliorated DOX-induced cardiotoxicity by reducing oxidative stress (5). In our study the monoterpene R-(-)-carvone protected the heart *in vivo* by increasing catalase activity, while it synergistically enhanced the cytotoxic action of DOX on the MCF 7 cancer cell line.

In conclusion, R-(-)-carvone exerted a cardioprotective effect through increasing catalase activity while enhancing the cytotoxicity of DOX on the breast cancer cell line. Future investigations are needed to explore the mechanism of the synergistic effect of R-(-)-carvone with DOX on breast cancer and other different cancer cell lines. Also, clinical studies are required to determine the efficacy of R-(-)-carvone as an adjuvant in cancer therapy, its optimal dose, and effectiveness in a specific cancer types (18).

## Figures and Tables

**Table 1:**
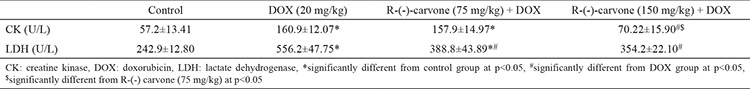
Results of biochemical tests (mean ± SEM) of mice treated with vehicle, doxorubicin, R-(-)-carvone or R-(-)-carvone with doxorubicin

**Table 2:**
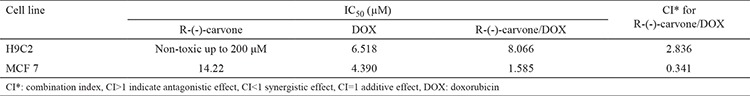
IC_50_ (μM) of R-(-)-carvone, doxorubicin and R-(-)-carvone/doxorubicin combination in studied cell lines and their combination indices according to CompuSyn

## References

[1] Osman AM, Al-Harthi SE, AlArabi OM, Elshal MF, Ramadan WS, Alaama MN, et al (2013). Chemosensetizing and cardioprotective effects of resveratrol in doxorubicin- treated animals. Cancer Cell Int.

[2] Zeiss CJ, Gatti DM, Toro-Salazar O, Davis C, Lutz CM, Spinale F, et al (2019). Doxorubicin-induced cardiotoxicity in collaborative cross (cc) mice recapitulates individual cardiotoxicity in humans. G3: (Bethesda).

[3] Hanusova V, Bousova I, Skalova L (2011). Possibilities to increase the effectiveness of doxorubicin in cancer cells killing. Drug Metab Rev.

[4] Osman HE, Abohassan AA (2012). Morphological and Analytical characterization of Moringa peregrina Populations In Western Saudi Arabia. IJTAS.

[5] Trivedi PP, Kushwaha S, Tripathi DN, Jena GB (2011). Cardioprotective effects of hesperetin against doxorubicin-induced oxidative stress and DNA damage in rat. Cardiovasc Toxicol.

[6] Sarkic A, Stappen I (2018). Essential oils and their single compounds in cosmetics—a critical review. Cosmetics.

[7] Vinothkumar R, Sudha M, Viswanathan P, Kabalimoorthy J, Balasubramanian T, Nalini N (2013). Modulating effect of d-carvone on 1,2-dimethylhydrazine-induced pre-neoplastic lesions, oxidative stress and biotransforming enzymes, in an experimental model of rat colon carcinogenesis. Cell Prolif.

[8] Wattenberg LW, Sparnins VL, Barany G (1989). Inhibition of N-nitrosodiethylamine carcinogenesis in mice by naturally occurring organosulfur compounds and monoterpenes. Cancer Res.

[9] Cinici E, Dilekmen N, Kutlu Z, Dincer B, Cinici O, Balta H, et al (2019). Carvone protects against paclitaxel-induced retinal and optic nerve cytotoxicity: a histopathological study. Cutan Ocul Toxicol.

[10] Antoniak S, Tatsumi K, Schmedes CM, Grover SP, Pawlinski R, Mackman N (2018). Protease-activated receptor 1 activation enhances doxorubicin-induced cardiotoxicity. J Mol Cell Cardiol.

[11] Lowry OH, Rosebrough NJ, Farr AL, Randall RJ (1951). Protein measurement with the Folin phenol reagent. J Biol Chem.

[12] Chou T-C, Talalay P (1984). Quantitative analysis of dose-effect relationships: the combined effects of multiple drugs or enzyme inhibitors. Adv Enzyme regul.

[13] Liu Z, Shi A, Song D, Han B, Zhang Z, Ma L, et al (2017). Resistin confers resistance to doxorubicin-induced apoptosis in human breast cancer cells through autophagy induction. Am J Cancer Res.

[14] Bicas J, Neri-Numa I, Ruiz A, De Carvalho J, Pastore G (2011). Evaluation of the antioxidant and antiproliferative potential of bioflavors. Food Chem Toxicol.

[15] Patel PB, Thakkar VR (2014). L-carvone induces p53, caspase 3 mediated apoptosis and inhibits the migration of breast cancer cell lines. Nutr Cancer.

[16] Jaafari A, Tilaoui M, Mouse HA, M'bark LA, Aboufatima R, Chait A, et al (2012). Comparative study of the antitumor effect of natural monoterpenes: relationship to cell cycle analysis. Rev Bras Farmacogn.

[17] Lee SK (2018). Sex as an important biological variable in biomedical research. BMB Rep.

[18] Fatima S (2018.). Cancer Treatment with Pro and. Antioxidant Agents.

[19] dos Santos DS, dos Santos Goldenberg RC (2018.). Doxorubicin-Induced Cardiotoxicity: From Mechanisms to Development of Efficient Therapy. Cardiotoxicity: IntechOpen.

[20] Singh K, Bhori M, Kasu YA, Bhat G, Marar T (2018). Antioxidants as precision weapons in war against cancer chemotherapy induced toxicity-Exploring the armoury of obscurity. SPJ.

[21] Marinello PC, Panis C, Silva TNX, Binato R, Abdelhay E, Rodrigues JA, et al (2019). Metformin prevention of doxorubicin resistance in MCF-7 and MDA-MB-231 involves oxidative stress generation and modulation of cell adaptation genes. Sci Rep..

[22] Kang YJ, Chen Y, Epstein PN (1996). Suppression of doxorubicin cardiotoxicity by overexpression of catalase in the heart of transgenic mice. J Biol Chem.

[23] Wu Z, Tan B, Liu Y, Dunn J, Martorell Guerola P, Tortajada M, et al (2019). Chemical Composition and Antioxidant Properties of Essential Oils from Peppermint, Native Spearmint and Scotch Spearmint. Molecules.

[24] Rehman MU, Tahir M, Khan AQ, Khan R, Lateef A, et al (2014). D-limonene suppresses doxorubicin-induced oxidative stress and inflammation via repression of COX-2, iNOS, and NFκB in kidneys of Wistar rats. Exp Biol Med.

[25] Crespo R, Wei K, Rodenak-Kladniew B, Mercola M, Ruiz-Lozano P, Hurtado C (2017). Effect of geraniol on rat cardiomyocytes and its potential use as a cardioprotective natural compound. Life Sci.

